# Liver fatty acid-binding protein (L-FABP) promotes cellular angiogenesis and migration in hepatocellular carcinoma

**DOI:** 10.18632/oncotarget.7571

**Published:** 2016-02-22

**Authors:** Chung-Yu Ku, Yu-Huei Liu, Hsuan-Yuan Lin, Shao-Chun Lu, Jung-Yaw Lin

**Affiliations:** ^1^ Institute of Biochemistry and Molecular Biology, College of Medicine, National Taiwan University, Taipei City, Taiwan; ^2^ Graduate Institute of Integrated Medicine, China Medical University, Taichung City, Taiwan; ^3^ Department of Life Science, National Taiwan Normal University, Taipei City, Taiwan

**Keywords:** angiogenesis, hepatocellular carcinoma, liver fatty acid-binding protein, vascular endothelial growth factor

## Abstract

Liver fatty acid-binding protein (L-FABP) is abundant in hepatocytes and known to be involved in lipid metabolism. Overexpression of L-FABP has been reported in various cancers; however, its role in hepatocellular carcinoma (HCC) remains unclear. In this study, we investigated L-FABP and its association with vascular endothelial growth factors (VEGFs) in 90 HCC patients. We found that L-FABP was highly expressed in their HCC tissues, and that this expression was positively correlated with that of VEGF-A. Additionally, L-FABP significantly promoted tumor growth and metastasis in a xenograft mouse model. We also assessed the mechanisms of L-FABP activity in tumorigenesis; L-FABP was found to associate with VEGFR2 on membrane rafts and subsequently activate the Akt/mTOR/P70S6K/4EBP1 and Src/FAK/cdc42 pathways, which resulted in up-regulation of VEGF-A accompanied by an increase in both angiogenic potential and migration activity. Our results thus suggest that L-FABP could be a potential target for HCC chemotherapy.

## INTRODUCTION

Hepatocellular carcinoma (HCC), the most common type of liver cancer, is notoriously resistant to systemic therapies and has a relatively high recurrence rate. The poor prognosis associated with HCC causes more than 700,000 deaths annually and has become the third leading cause of cancer-related death worldwide [1, 2]. Angiogenesis plays an important role in the progression and metastasis of HCC, and vascular endothelial growth factors (VEGFs) are critical drivers of the “angiogenic switch” in tumors, which is a process that stimulates the formation of new blood vessels to supply the nutrients and oxygen required for sustained tumor growth [1]. VEGF ligands bind to three similar receptor tyrosine kinases, namely VEGFR1 (FLT1), VEGFR2 (KDR), and VEGFR3 (FLT4), by different affinities; however, VEGFR2 is the major receptor for VEGF-induced signaling and therefore serves as a major therapeutic target [3]. Because HCC is often diagnosed at an advanced stage and is accompanied by tumor angiogenesis and metastasis, VEGF-targeted therapies may have therapeutic benefits [2, 4].

Liver fatty acid-binding protein (L-FABP), a member of the FABP family, is expressed abundantly in the cytoplasm and can bind hydrophobic lipid ligands with a high specificity. L-FABP uniquely binds two ligand molecules (long chain fatty acids) or various hydrophobic molecules (e.g., cholesterol and bile acids) [5]. Furthermore, L-FABP interacts with the plasma membrane to enhance cholesterol transfer or participate in membrane microdomain alteration [6]; however, the mechanisms underlying L-FABP activity are currently unclear.

Overexpression of L-FABP has been observed in various cancers, including liver, lung, gastric, and colon cancers. Moreover, several studies have indicated that L-FABP expression is correlated with VEGF expression in HCC [7, 8]. The precise mechanisms underlying this correlation remain unknown; therefore, in this study, we investigated the association between L-FABP and VEGF in 90 HCC patients. We found that L-FABP was highly expressed in the tumor tissue of these patients compared with expression in their normal adjacent tissue, and we observed a positive correlation between L-FABP and VEGF-A expression. Overall, our study suggests that L-FABP participates in HCC malignancy and could serve as a potential target for HCC therapy.

## RESULTS

### Overexpression between L-FABP and VEGF-A in HCC tissues is positively correlated

**Figure 1:**
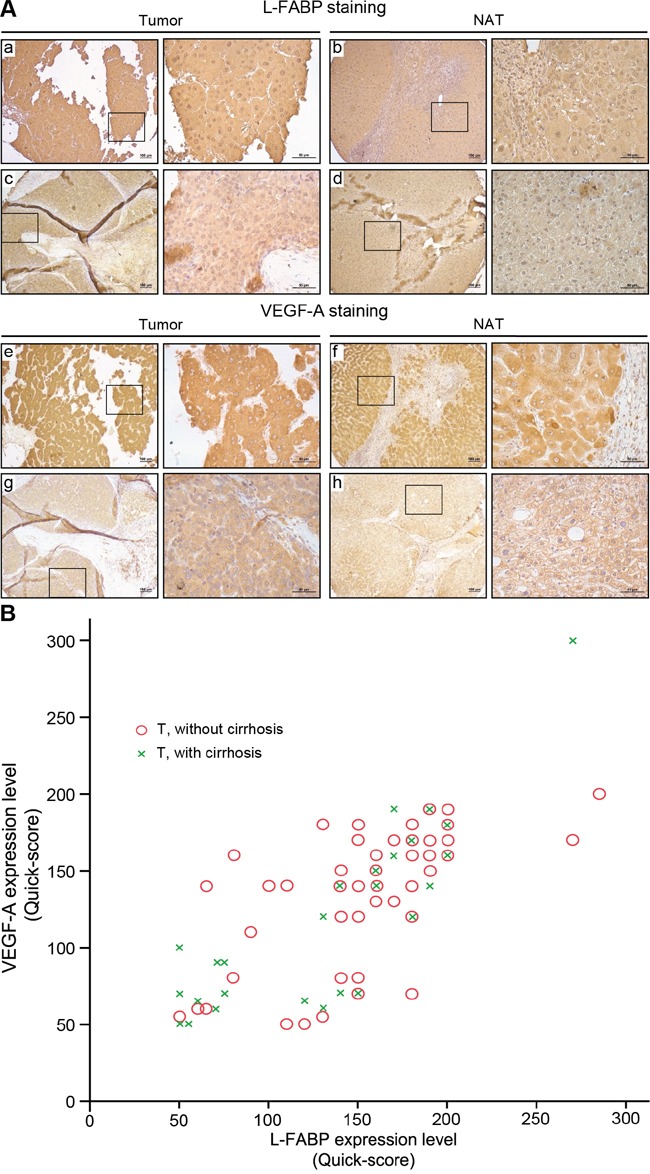
Expression of L-FABP and VEGF-A in tissues obtained from HCC patients Protein expression was assessed in 90 HCC cases using immunohistochemical staining of paired normal (NAT) and tumor tissues. **A.** Staining of L-FABP and VEGF-A was observed in tumor tissues (L-FABP: a and c; VEGF-A: e and g) and their paired normal adjacent tissues (L-FABP: b and d; VEGF-A: f and h). Staining intensity: a and e, strong; b, c, f, and g, moderate; d and h, weak. **B.** Positive correlation between L-FABP and VEGF-A expression in 90 HCC tissues with and without cirrhosis (Pearson's correlation coefficient, r = 0.737; p < 0.01).

Following immunohistochemical staining, expression levels of L-FABP in 90 pairs of tissue (HCC tumor and normal adjacent tissue) were classified by staining intensity as negative, weak, moderate, or strong; the associated photographs are shown in Figure 1A. Expression of L-FABP was significantly higher in all tumor tissues (HCC with or without cirrhosis) compared with NAT (Table 1, p = 0.012). In addition, the expression of L-FABP was positively correlated with that of VEGF-A (Pearson correlation: r = 0.737, p < 0.01, n = 90) (Figure 1B). Thus, up-regulation of L-FABP is apparently correlated with HCC VEGF-A expression.

**Table 1:** Correlation between L-FABP and VEGF-A protein expression in tissue pairs from 90 HCC patients

Intensity ^a^		NAT, N (%)	HCC without cirrhosis, N (%)	HCC with cirrhosis, N (%)	P value ^b^	P value ^c^	P value ^d^	P value ^e^	P value ^f^
L-FABP	1	15 (44.1)	8 (23.5)	11 (32.4)	0.012	0.028	0.027	0.040	0.086
	2	72 (55.0)	40 (30.5)	19 (14.5)					
	3	3 (20.0)	9 (60.0)	3 (20.0)					
VEGF-A	1	25 (48.1)	12 (23.1)	15 (28.8)	0.025	0.563	0.360	0.037	0.017
	2	65 (51.2)	45 (35.4)	17 (13.4)					
	4	0 (0.0)	0 (0.0)	1 (100.0)					

Abbreviations: HCC, hepatocellular carcinoma; OR, odds ratios; CI, confidence interval; N, number.

a Intensity: 0, negative; 1, weak positive; 2, moderate positive; 3, strong positive; 4, very strong positive.

b Chi-square test, NAT vs HCC without cirrhosis vs HCC with cirrhosis.

c Chi-square test, NAT vs HCC with or without cirrhosis.

d Chi-square test, NAT vs HCC without cirrhosis.

e Chi-square test, NAT vs HCC with cirrhosis.

f Chi-square test, HCC without cirrhosis vs HCC with cirrhosis.

### L-FABP induces VEGF-A expression and increases angiogenic potential in immortalized Hus and Huh7 cells

**Figure 2:**
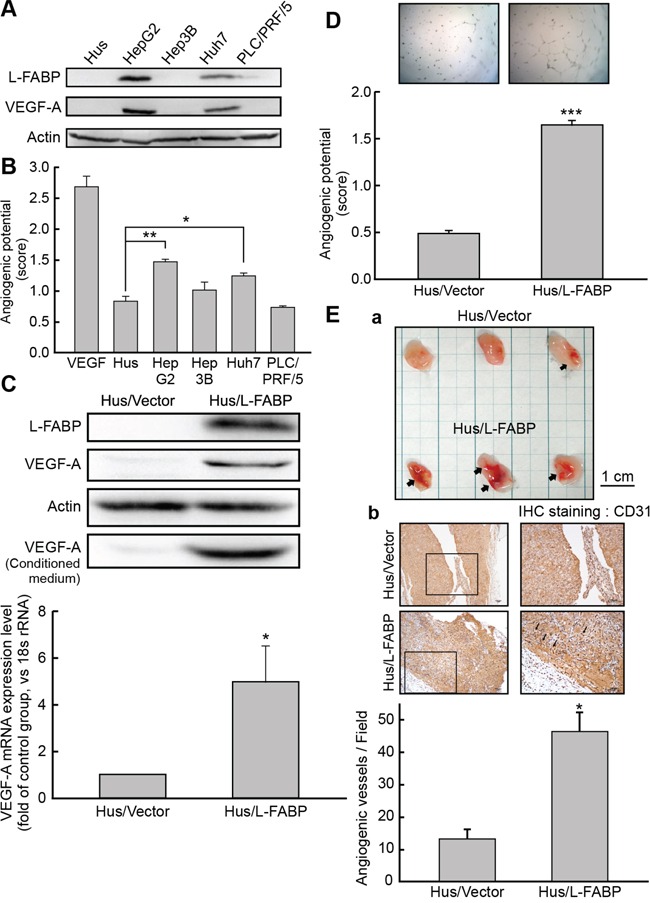
L-FABP promotes VEGF-A expression and angiogenic activity of liver cells **A.** Western blot analysis for L-FABP expression in normal immortalized hepatocyte (Hus) and hepatocellular carcinoma (HepG2, Hep3B, Huh7 and PLC/PRF/5) cell lines. **B.** Angiogenic potential (score: see *In vitro tube formation assay* in Methods for details) of Hus, HepG2, Hep3B, Huh7, and PLC/PRF/5 cells. ***p < 0.001 versus Hus cells. **C.** Western blotting analysis of L-FABP and VEGF-A expression in Hus/L-FABP and Hus/Vector (vector-only control) cells. *p < 0.05 versus Hus/Vector control. **D.** *In vitro* angiogenic potential (score: see panel B) of Hus/L-FABP and Hus/Vector cells. Angiogenic vascular tube was imaged at 8 h. ***p < 0.001 versus Hus/Vector control. **E.** *In vivo* angiogenic activity of Hus/L-FABP and Hus/Vector cells assessed using a Matrigel plug assay. a: Matrigel plugs recovered from mice injected with Hus/Vector and Hus/L-FABP cells. Arrows indicate infiltration of blood vessels. b: Immunohistochemical (IHC) staining of CD31 (angiogenesis marker) in Matrigel plugs showed that Hus/L-FABP promoted angiogenesis, and the positively stained vessels are indicated by arrows. *p < 0.05 versus Hus/Vector control (n = 3).

L-FABP expression was analyzed in various cell lines, including Hus (normal hepatocytes) and HCC (HepG2, Hep3B, Huh7, and PLC/PRF/5) cells. L-FABP was highly expressed in HepG2 and Huh7 cells, which also showed strong VEGF-A expression levels in their cytosol (Figure 2A) and culture medium (Supplementary Figure 1), in addition to higher angiogenic potential (Figure 2B).

To examine the effects of L-FABP on VEGF-A expression, we generated L-FABP-overexpressing stable clones with Hus cells and we used Huh7 cells to produce L-FABP shRNA knockdown clones. As shown in Figure 2C, VEGF-A expression (at both the mRNA and protein levels) was higher in Hus/L-FABP cells than in control cells, whereas the expression of VEGF-A decreased markedly in Huh7/L-FABP shRNA cells relative to the control (Supplementary Figure 2A).

Angiogenesis was also significantly higher in Hus/L-FABP cells than in control cells (Figure 2D), i.e., it decreased in Huh7/L-FABP shRNA cells (Supplementary Figure 2B). To further examine whether L-FABP promotes angiogenesis *in vivo*, we performed a Matrigel plug assay in NOD/SCID mice by using Hus/L-FABP (Figure 2E, a) or Huh7/L-FABP shRNA cells (Supplementary Figure 2C). Anti-CD31 immunohistochemical staining indicated that L-FABP-overexpressed cells promoted angiogenesis by inducing neovascular formation in Matrigel (Figure 2E, b, p < 0.05).

### L-FABP interacts with VEGFR2 in membrane rafts

**Figure 3:**
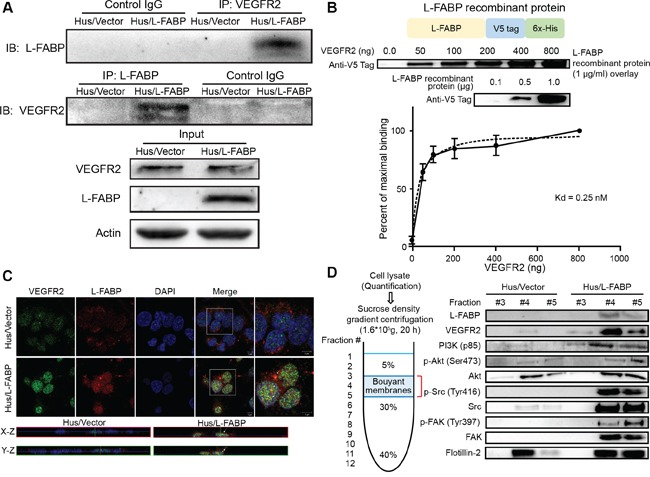
L-FABP associates with VEGFR2 on membrane rafts **A.** Left: Hus/L-FABP and Hus/Vector (vector-only control) cells were subjected to either immunoprecipitation (IP) with a VEGFR2 antibody followed by blotting with L-FABP or IP with an L-FABP antibody followed by blotting with VEGFR2. Right: Cell lysates (50 μg) were immunoblotted as an input control. **B.** L-FABP/V5-tagged recombinant protein was purified by Ni-NTA resin and subjected to SDS-PAGE to determine the purity as shown in the top photo. The overlay assay (far western blot analysis) was performed to estimate the affinity of the interaction between the VEGFR2 intracellular domain and L-FABP. PVDF membranes containing 0.5 to 8.0 μg of VEGFR2 recombinant protein (aa 789 to end) were incubated with L-FABP/V5-tagged recombinant protein (1 μg/ml) for 12 hours. The specific binding between L-FABP and VEGFR2 increased obviously between 0 to 2 μg, and maximal binding was observed at 8μg. The binding observed at the other concentrations was expressed as a percentage of the maximal binding within each experiment, and the Kd for binding between L-FABP and VEGFR2 was calculated as 0.25 nM. **C.** Three-color confocal images of cells that were fixed and stained with antibodies against L-FABP and VEGFR2. Signals: green, VEGFR2-Alexa 488; red, L-FABP-Alexa 568; and blue, DAPI. Magnification: 63×. The bottom photos show the X-Z and Y-Z optical sections, respectively, of Hus/Vector and Hus/L-FABP cells. Arrows indicate the co-localization of VEGFR2 and L-FABP in the apical membrane. **D.** Membrane localization of L-FABP, VEGFR2, PI3K (p85), phospho-Akt (Ser473), Akt, phosho-Src (Tyr416), Src, FAK, and phosho-FAK (Tyr397) in Hus/L-FABP or Hus/Vector cells. Membrane rafts were determined by using sucrose gradient-based ultracentrifugation and analyzed with western blotting (fraction #3–5).

Previous studies reported that some FABPs, such as H-FABP or B-FABP, could interact with membrane-associated receptors, including integrin or dopamine D2 receptor [9–11], and that L-FABP may associate with cell membranes or membrane proteins [5, 12]. A recent study also revealed that L-FABP was significantly associated with hepatocyte plasma membrane cholesterol-rich microdomains [13]. Thus, we examined whether L-FABP interacted with membrane receptors as follows. We proposed that L-FABP was associated with membrane receptors and, by using an alignment of amino acid sequences thought to interact with FABP, we identified the consensus sequence most likely to interact with L-FABP: WKIGFXKRLXXVXXXI (Supplementary Figure 3). By comparing the consensus sequence to that of membrane receptors, we found that the kinase domain of VEGFR2 potentially interacted with L-FABP. A co-immunoprecipitation assay with primary antibodies against VEGFR2 or L-FABP, in addition to blotting with L-FABP or VEGFR2, showed that L-FABP could interact with VEGFR2 (Figure 3A). To further confirm this result, we purified L-FABP recombinant protein using a nickel column (Supplementary Figure 4), and performed an overlay assay (far-western blot analysis) to study the interaction between L-FABP and VEGFR2. We found that L-FABP directly interacted with the VEGFR2 intracellular domain (aa 789 to end) in a cell-free system. Moreover, the binding curve revealed that the Kd (dissociation constant) for the interaction of VEGFR2 and L-FABP was 0.25 nM (Figure 3B).

Confocal microscopy images of Hus/L-FABP cells revealed that L-FABP was located in both the membrane and cytosol, whereas VEGFR2 was expressed mainly at the membrane. Co-localization of L-FABP and VEGFR2 in the apical membrane was observed in Hus/L-FABP cells (Figure 3C), but disappeared in Huh7 cells with L-FABP knockdown (Supplementary Figure 5). Sucrose gradient ultracentrifugation was conducted to confirm this co-localization. Fractions with lipid rafts of Hus/L-FABP cells were identified by flotillin-2, a lipid raft marker; moreover, not only L-FABP and VEGFR2 but also membrane associating signal transduction proteins (e.g., PI3K (p85), p-Akt/Akt, p-Src/Src, p-FAK/FAK) showed increased distribution in membrane rafts (Figure 3D). Taken together, these results suggest that overexpressed L-FABP was not only associated with membrane VEGFR2 but also activated its downstream signal transduction.

### L-FABP increases VEGFR2/Src phosphorylation and cell migration via the FAK/cdc42 pathway

**Figure 4:**
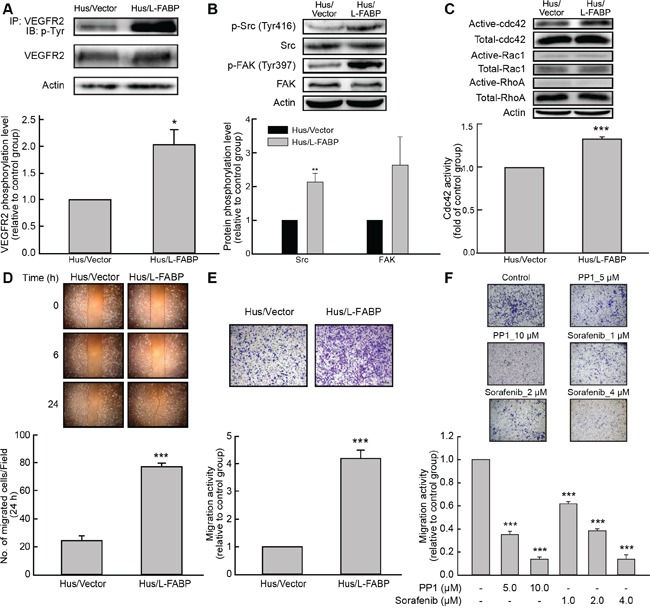
L-FABP increases cell migration activity via VEGFR2/Src signaling and the FAK/cdc42 pathway **A.** Phosphorylation of VEGFR2 in Hus/L-FABP and Hus/Vector (vector-only control) cells assessed by immunoprecipitation (IP) with a VEGFR2 antibody and blotting with a phospho-tyrosine antibody. *p < 0.05 versus Hus/Vector control. **B.** Phosphorylation of Src (Tyr416) and FAK (Tyr397) in Hus/L-FABP and Hus/Vector cells analyzed by western blotting. **p < 0.01 versus Hus/Vector control. **C.** Small GTPase binding in Hus/L-FABP or Hus/Vector cells. Active cdc42 and Rac1, but not RhoA, were detected by western blotting analysis. ***p < 0.001 for cdc42 activity versus Hus/Vector control. **D.** Wound-healing migration (2D migration activity) of Hus/L-FABP and Hus/Vector cells over 24 h. ***p < 0.001 versus Hus/Vector control. **E.** Migration activity of Hus/L-FABP and Hus/Vector cells seeded onto Boyden chambers and allowed to migrate toward 10% serum-containing medium for 16 h. ***p < 0.001 versus Hus/Vector control. **F.** Migration activity of Hus/L-FABP cells treated with PP1 (Src inhibitor: 5 or 10 μM) or Sorafenib (VEGFR2 inhibitor: 1, 2, or 4 μM) for 16 h. ***p < 0.001 versus DMSO-treated control group.

The VEGFR2/Src pathway reportedly affects cancer cell migration by activating FAK and Rho-GTPase [14–16]. In Hus/L-FABP cells, the phosphorylation of VEGFR2, Src, and FAK increased significantly (Figure 4A and 4B) and the activity of cdc42 was significantly increased (Figure 4C, p < 0.001). A wound-healing assay (Figure 4D) and Boyden chamber-based migration assay (Figure 4E) of 2D and 3D migration activity, respectively, showed that migration activity was higher in Hus/L-FABP cells than in control cells. To determine whether or not L-FABP-induced cell migration occurred via cdc42, plasmids expressing different variants of cdc42, including wild-type (WT), constitutively active (CA), and dominant negative (DN) cdc42, were transfected into the cells. Phalloidin staining (Supplementary Figure 6A) and a transwell assay (Supplementary Figure 6B) showed that the activity of cdc42 strongly affects actin rearrangement and cell migration induced by L-FABP, which was in line with our previous findings. In contrast, L-FABP-knockdown clones showed significantly reduced 3D migration activity (Supplementary Figure 2D, p < 0.001). Sorafenib (VEGFR2 inhibitor) or PP1 (Src inhibitor) treatments significantly inhibited 3D migration activity in Hus/L-FABP cells (Figure 4F, p < 0.001). Moreover, knockdown of L-FABP in Hus/L-FABP-stable clones reversely decreased their 3D migration activity (Supplementary Figure 7C). These results suggest that VEGFR2/Src signaling participates in L-FABP-induced migration activity via the FAK/cdc42 pathway.

### L-FABP induces VEGF-A expression via the Akt/mTOR/P70S6K/4EBP1 pathway in a HIF-1α-dependent manner

**Figure 5:**
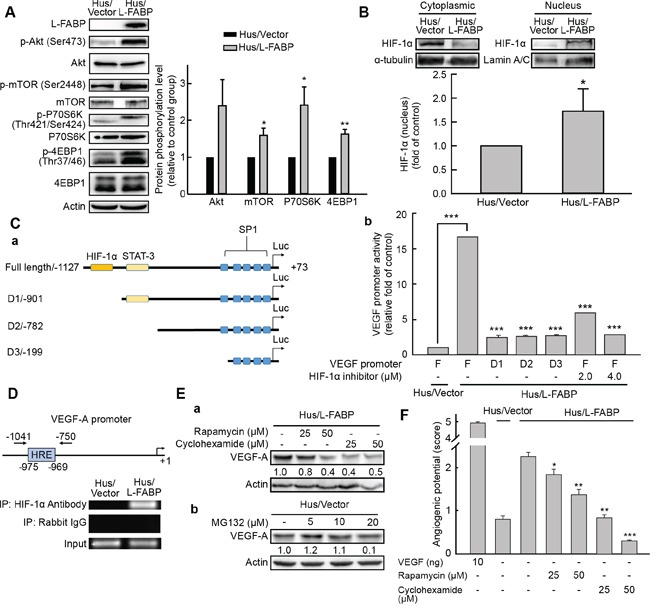
L-FABP-promoted VEGF-A expression is regulated by HIF-1α via the Akt/mTOR/P70S6K/4EBP1 pathway **A.** Phosphorylation of Akt (Ser473), mTOR (Ser2448), P70S6K (Thr421/Ser424), and 4EBP1 (Thr37/46) in Hus/L-FABP and Hus/Vector (vector-only control) cells analyzed by western blotting. *p < 0.05 and **p < 0.01 versus Hus/Vector control. **B.** Nucleus and cytoplasmic localization of HIF-1α in Hus/L-FABP cells. Loading controls were α-tubulin and lamin A/C for cytoplasm and nucleus, respectively. HIF-1α levels increased ~1.7-fold in Hus/L-FABP relative to the control group: *p < 0.05. **C.** a: Diagram of the receptor constructs for the full-length VEGF-A promoter and deletion mutants (D1-D3). b: The luciferase activity of cell extracts was analyzed using a luciferase reporter assay (bar graph). ***p < 0.001 versus Hus/Vector control. **D.** Chromatin immunoprecipitation assay was performed to determine the amount of HIF-1α binding to the VEGF-A promoter; rabbit IgG served as a negative control, and the input served as a positive control. **E.** a: Western blot analysis of Hus/L-FABP cells treated with rapamycin (mTOR inhibitor: 25 or 50 μM) or cyclohexamide (translation inhibitor: 25 or 50 μM) for 12 h. b: Hus/Vector cells treated with MG132 (proteasome inhibitor: 5, 10, or 20 μM) for 24 h. **F.** *In vitro* angiogenic activity (score: see *In vitro tube formation assay* in Methods for details) of Hus/L-FABP and Hus/Vector cells treated with rapamycin or cyclohexamide (doses identical to those in D) for 12 h. ***p < 0.001 versus Hus/Vector control.

Based on the results shown in Figure 3D, and previous studies suggesting that Akt activation increases VEGF-A expression and is necessary and sufficient to regulate HIF-1α and VEGF expression in various human cancer cells [17–19], we postulated that the signal transduction of L-FABP-mediated VEGF-A expression was activated through the Akt pathway. In western blot analysis, we observed activation of the Akt/mTOR/P70S6K/4EBP1 pathway in Hus/L-FABP cells (Figure 5A). The expression of VEGF-A mRNA can apparently be regulated in an HIF-1α-dependent manner [20, 21]. Here, VEGF-A mRNA expression was significantly increased in L-FABP-overexpressing cells as mentioned in Figure 2C, and HIF-1α levels significantly increased in the nucleus fraction of Hus/L-FABP cells (Figure 5B, p < 0.05). Inhibition of the PI3K/Akt pathway by treatment with LY294002 (PI3K inhibitor) decreased the expression of HIF-1α and VEGF-A (Supplementary Figure 8). These data suggest that L-FABP induced VEGF-A expression through Akt activation, and that this process could be regulated by HIF-1α.

To confirm our findings, full-length and successive 5′ deletion (D1–D3) constructs of the VEGF-A gene promoter were cloned into pGL4.22 luciferase reporter vectors, and a luciferase reporter assay was conducted (Figure 5C, a). Results showed that VEGF-A transcriptional activity was elevated ~16.5-fold in L-FABP-overexpressing Hus cells compared with that in control cells, whereas deletion of the HIF-1α binding site (D1-D3) reduced this activity to ~2.5 fold that of the control (Figure 5C, b). Additionally, the chromatin immunoprecipitation assay demonstrated that the association between HIF-1α and the VEGF-A promoter was enhanced in Hus/L-FABP cells (Figure 5D). To further address the regulation of VEGF-A expression, Hus/L-FABP cells were treated with rapamycin (mTOR inhibitor) or cyclohexamide (translation inhibitor); consequently, decreased VEGF-A expression (Figure 5E, a) and concentration-dependent inhibition of angiogenic potential (Figure 5F) were observed. Treatment of Hus/Vector cells with the proteasome inhibitor MG132 also indicated that L-FABP-induced VEGF-A expression did not occur via inhibition of protein degradation (Figure 5E, b). Taken together, these data suggest that L-FABP-induced VEGF-A expression was regulated via the Akt/mTOR/P70S6K/4EBP1 pathway in a HIF-1α-dependent manner.

### L-FABP promotes tumor growth and metastasis *in vivo*

**Figure 6:**
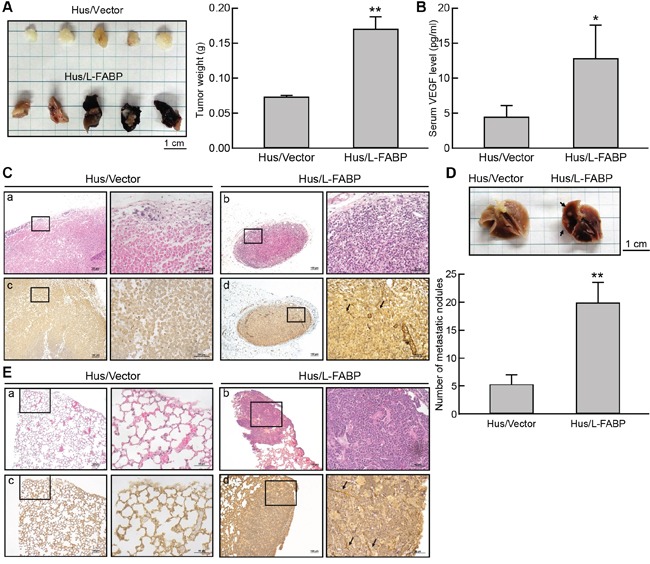
L-FABP promotes tumor growth and metastasis *in vivo* Hus/L-FABP or Hus/Vector (vector-only control) cells (2 × 10^6^) was subcutaneously injected into the hind limbs of NOD/SCID mice, and the resulting in situ tumors were removed after 8 weeks for analysis. **A.** Representative photograph and average weight of tumors (n = 5 per group). **p < 0.01 versus Hus/Vector control. **B.** VEGF-A content in the serum of treated mice. *p < 0.001 versus Hus/Vector control. **C.** Stained tumor sections from Hus/L-FABP- and Hus/Vector-injected mice. H&E staining (a and b) and anti-CD31 antibody immunohistochemical staining (c and d). The positive staining indicated by the arrows shows strong angiogenic activity in the Hus/L-FABP-injected group. **D.** Metastatic activity of Hus/L-FABP and Hus/Vector cells (5 × 10^6^) in a lung metastasis model (NOD/SCID mice). After 10 weeks, the lungs were excised from mice (see photograph), and metastatic nodules (indicated by arrows) were counted (n = 5 per group). **p < 0.01 versus Hus/Vector control. **E.** Angiogenesis activity in metastatic nodules was assessed via H&E staining (a and b) or anti-CD31 immunohistochemical staining (c and d), and positive staining is indicated by arrows.

The role of L-FABP in tumorigenesis was examined in immune-deficient NOD/SCID mice. At Day 50, tumor growth was significantly enhanced in mice injected with Hus/L-FABP cells, whereas no significant tumor growth was observed in Hus/Vector cell-injected (control) mice (Figure 6A). The level of VEGF-A in the serum of mice also increased 2.8-fold in the Hus/L-FABP group relative to the control group (Figure 6B). Additionally, immunohistochemical staining of CD31 indicated that L-FABP induced angiogenesis *in vivo* (Figure 6C). In an *in vivo* tumor metastasis assay, the number of metastatic nodules formed in the lungs of NOD/SCID mice after 60 days was 3.9-fold higher in the Hus/L-FABP-injected group relative to the control group (Figure 6D), and angiogenic vessel formation was increased in these nodules (Figure 6E). The results of these *in vivo* experiments support the correlation between L-FABP and VEGF-A expression.

### Cholesterol association and membrane interaction properties are essential for L-FABP-induced cell migration and angiogenesis

**Figure 7:**
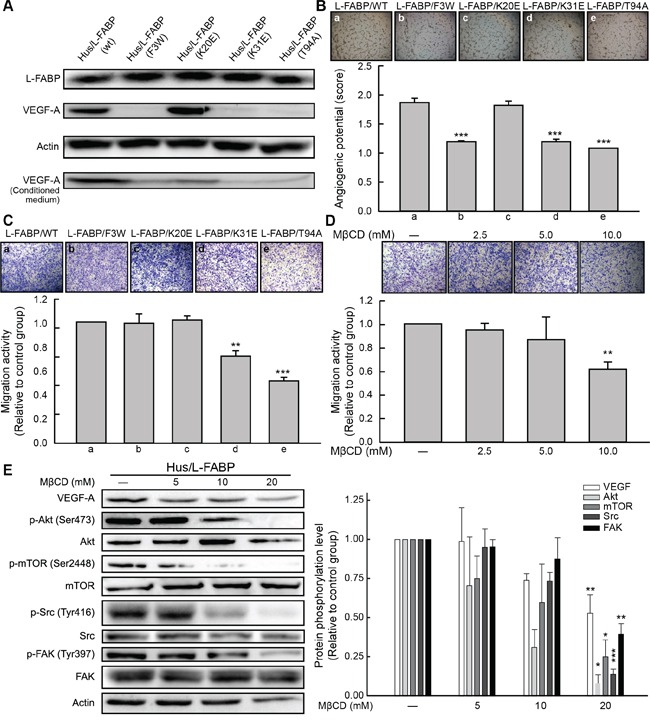
Cholesterol binding properties are essential for L-FABP-induced cell migration and angiogenesis **A.** Western blotting analysis of L-FABP and VEGF-A expression (both intracellular and extracellular levels) in various mutants of L-FABP-overexpressed stable cells generated by site-directed mutagenesis **B.** *In vitro* angiogenic activity (score: see *In vitro tube formation assay* in Methods for details) of mutants. Images represent amino acid substitutions: (a) L-FABP (wild type), (b) L-FABP (F3 to W), (c) L-FABP (K20 to E), (d) L-FABP (K31 to E), and (e) L-FABP (T94 to A). ***p < 0.001 versus wild-type. **C.** Migration activity of the mutants. Images (a–e) represent the amino acid substitutions described in (B). **p < 0.01, ***p < 0.001 versus wild-type. **D.** Migration activity of Hus/L-FABP cells treated with MβCD (cholesterol depletion agent: 5, 10, or 20 mM) for 12 h. **p < 0.01 versus water-treated control group. **E.** Western blot analysis of Hus/L-FABP cells treated with MβCD (5, 10, or 20 mM) for 6 h. *p < 0.05, **p < 0.01, ***p < 0.001 versus water-treated control group.

Previous studies suggested that L-FABP mutations prevent fatty acid or cholesterol uptake and even alter membrane structure [22–26]. Thus, to investigate the effects of L-FABP on the membrane of L-FABP-overexpressing cells, we used site-directed mutagenesis to generate L-FABP-overexpressing stable clones with substitutions of various functional amino acids in Hus cells. Three mutants, namely F3W, K31E, and T94A, exhibited reduced VEGF-A expression relative to the wild-type (Figure 7A), and these mutants showed significantly decreased angiogenic potential (Figure 7B). However, migration activity was significantly reduced in K31E and T94A mutants only (Figure 7C, p < 0.01). T94A is the most common mutation in Europeans, and it is known to affect fatty acid and cholesterol uptake as a loss-of-function mutation [24, 26]. For further substantiation, we reduced the membrane cholesterol content in Hus/L-FABP cells by using MβCD (a cholesterol depletion reagent). VEGF expression, migration activity, and related signaling were all down-regulated in MβCD-treated Hus/L-FABP cells (Figure 7D and 7E). Overall, our data suggest that the oncogenic activity of L-FABP was associated with its membrane-binding properties.

## DISCUSSION

HCC is characterized by its aggressiveness and angiogenic capability; thus, the angiogenic factor VEGF is considered to be a target for HCC therapy [1, 3]. Here, we reported for the first time that overexpression of L-FABP plays an important role in VEGF-A expression and cell migration in HCC. Furthermore, we demonstrated that L-FABP associates with VEGFR2 in the cell membrane, which leads to activation of VEGFR2-related signaling (i.e., Src/FAK/cdc42 and Akt/mTOR/HIF-1α signaling). Additionally, we showed that the T94A mutation of L-FABP, which is related to cholesterol association activity, significantly decreases the angiogenic potential and migration activity of L-FABP-overexpressing cells.

It has been suggested that L-FABP promotes the growth of hepatocytes and protects cells from ROS via its anti-oxidative activity, which is related to methionine and cysteine [27, 28]. Other studies have also found evidence for a correlation between L-FABP and VEGF [7, 8]. A recent study reported that FABP4 (A-FABP) plays an important role in regulating the function of VEGF function and promoting proliferation of HUVEC cells [29]; however, the link between L-FABP and tumor malignancy is still unclear. Here, we found a significant increase in L-FABP expression in tumor tissue relative to normal adjacent tissue in 90 HCC patients, and L-FABP and VEGF-A expression was positively correlated in these tissues. L-FABP and VEGF-A expression was also higher in malignant HCC cell lines (HepG2 and Huh7) than in immortalized normal hepatocytes (Hus cells). Given these findings, we propose that L-FABP may participate in VEGF-A expression in HCC. Further investigation supported this hypothesis, with stable clones of Hus/L-FABP cells exhibiting increased VEGF-A expression and angiogenic potential both *in vitro* and *in vivo*. Additional evidence was provided by our experiments involving L-FABP knockdown in Huh7 cells, L-FABP-overexpressing Hus cells (Supplementary Figure 7A and 7B), and HepG2 cells (Supplementary Figure 9A). A previous study suggested that VEGF is essential for HCC cell migration [30]. We found that the migration activity of Hus/L-FABP cells increased significantly compared with that of control cells. Knockdown of L-FABP in Huh7, L-FABP-overexpressing Hus cells, and HepG2 cells (Supplementary Figure 9B) significantly decreased migration activity relative to control groups. Taken together, these results suggest that L-FABP overexpression plays a critical role in the angiogenic potential and migration activity of HCC cells, and that this effect can be reversely regulated using RNA knockdown technology.

Previous studies have clarified the binding mechanism of VEGF-A to VEGFR2, which consists of one VEGF-A dimer binding to one VEGFR2 homo- or hetero-dimer. The Kd of VEGF-A and VEGFR2 was also calculated by different methods (for ITC, Kd = 18 ± 5.2 nM, for SPR, Kd = 36.7 ± 5.9 nM) [31]. In the present study, we found that L-FABP interacts with VEGFR2 in cells overexpressing L-FABP. We also determined that the Kd of L-FABP and VEGFR2 (intracellular domain) in a cell-free system was 0.25 nM, suggesting that L-FABP could be a cytosolic interacting protein for VEGFR2. Because the detailed binding mechanism remains unclear, further studies should be performed using the SPR system (Biacore) or protein crystallization.

In an early study, L-FABP was reported to interact with the cell membrane [6]. However, most subsequent studies have focused on its biological function in transporting fatty acids and regulating lipid metabolism [32]. Previously, L-FABP was found to be co-expressed with VEGF in the cell membrane [8], and other studies have suggested that lipid rafts are capable of acting in signaling platforms [33–35]. In a similar manner, our confocal microscopy images showed that L-FABP and VEGFR2 co-localized on the cell surface. Furthermore, levels of downstream signaling proteins, including Src/FAK and PI3K/Akt, increased in the membrane fraction. Knockdown of L-FABP and VEGFR2 in Hus/L-FABP cells decreased the phosphorylation of these downstream signal molecules (Supplementary Figure 7D and 10). Further results also suggested that L-FABP was required for promotion of VEGFR2 activity (Supplementary Figure 11). Taken together, our findings provide evidence that L-FABP activates VEGFR2 signaling in HCC cells.

The regulation of VEGF in HCC has been highlighted because the related pathway plays an important role in cancer progression [2]. Indeed, only anti-VEGFR2 therapy has provided a significant benefit to clinical HCC patients and been approved by the FDA [4]. Here, we found that VEGF-A expression was regulated by the PI3K/Akt pathway and HIF-1α, as the transcriptional activity of VEGF-A was significantly increased by L-FABP overexpression and reduced by deletion of the HIF-1α binding site on the VEGF-A promoter. The higher level of secreted VEGF-A observed using the human growth factor array further supported our findings (Supplementary Figure 12). Interestingly, a previous study also showed that L-FABP levels were positively correlated with levels of VEGF-A mRNA [7]. When considered together, these results suggest that L-FABP may regulate VEGF-A expression in HCC cells via the PI3K/Akt pathway in a HIF-1α-dependent manner.

In previous studies, L-FABP-knockout mice showed decreased lipid metabolism and increased incidence of the obese phenotype with a high-fat diet [32, 36]. Mutation studies revealed that the binding of L-FABP to phospholipids was significantly decreased in the Phe3-to-Trp mutant, and Lys31 contributed to phospholipid binding [22, 23]. Other studies have also suggested that the T94A mutation alters the structure and stability of L-FABP and causes a loss of function [24–26]. In our experiment, F3W, K31E, and T94A mutations exhibited decreased levels of VEGF and reduced angiogenic potential; however, reduced migration activity was observed in K31E and T94A only. The effects of a membrane cholesterol depletion agent (MβCD) [37] and cholesterol-lowering compounds (filipin and simvastatin) [38] (Supplementary Figure 13) further supported the findings of the mutation investigation, with results suggesting that the cholesterol-associating activity of L-FABP was important for its function. The T94A mutation of L-FABP further disrupts the interaction of L-FABP and VEGFR2 (Supplementary Figure 14). Although the precise mechanisms still need further investigation, these findings indicate that the function of L-FABP in the cell membrane is related not only to metabolism but also to oncogenic potential.

HCC is a highly heterogeneous disease displaying differences in angiogenesis, extracellular matrix proteins, and tumor cell microenvironment [39]. Because VEGF levels are correlated with HCC malignancy and poor prognosis [40], previous studies focused on angiogenic heterogeneity, especially the relationship of VEGF and HIF-1α expression to HCC [4]. Whether or not L-FABP and VEGF-A, in addition to other onco-proteins, also show correlated expression in benign hepatocyte cell lines (e.g., Hus and Chang) or HCC cell lines is an interesting question, but little information is currently available. Furthermore, the copy number or methylation status of genes of interest should also be analyzed in future studies. A recent study revealed that L-FABP promotes diet-induced fatty liver disease and hepatic steatosis [41], but L-FABP expression was not significantly correlated with clinical pathologic characteristics (including age, sex, grade, invasion, metastasis, and stage) in the present study (Table 2). However, we found that L-FABP was significantly up-regulated in HCC patients with and without cirrhosis. Moreover, in the cirrhosis patients, high L-FABP expression was related to a higher risk of poor survival (Supplementary, Figure 15). Previous studies suggested that the “angiogenic switch” is required for the formation of a solid HCC tumor [42], and that VEGF is involved in an autocrine feed-forward loop that triggers angiogenesis [43, 44]. Because the relationship between L-FABP expression and HCC progression remains unclear, and because there is no appropriate prognosis marker for HCC with cirrhosis [45], L-FABP may serve as a potential research target in further studies.

**Table 2:** Association of L-FABP protein expression with clinical pathologic characteristics in patients with HCC

Characteristics	Low (1)	Intermediate (2)	High (3)	P value
**Age (years) Mean ±SD**	53.2 ± 8.5	52.4 ± 10.6	59.3 ± 8.2	0.089 ^a^
**Age >= 53.5**	9 (47.4)	30 (51.7)	8 (66.7)	0.555 ^b^
**Sex**				
Female	4 (21.1)	7 (11.9)	1 (8.3)	0.509 ^b^
Male	15 (78.9)	52 (88.1)	11 (91.7)	
Grade				0.484 ^b^
G1	2 (10.5)	3 (5.1)	1 (8.3)	
G2	15 (78.5)	46 (78.0)	7 (58.3)	
G3	2 (10.5)	10 (16.9)	4 (33.3)	
**pT (invasion depth)**				0.169 ^b^
T1	2 (10.5)	8 (14.3)	2 (16.7)	
T2	9 (47.4)	20 (35.7)	2 (16.7)	
T3	6 (31.6)	28 (50.0)	7 (58.3)	
T4	2 (10.5)	0 (0.0)	1 (8.3)	
**pN (lymph node metastasis)**				0.759 ^b^
N0	19 (100.0)	54 (98.2)	11 (100.0)	
N1	0 (0.0)	1 (1.8)	0 (0.0)	
**pM (distant metastasis)**				0.578 ^b^
M0	19 (100.0)	54 (96.4)	11 (100.0)	
M1	0 (0.0)	2 (3.6)	0 (0.0)	
**TNM stage**				0.546 ^b^
I	2 (10.5)	8 (14.3)	2 (16.7)	
II	9 (47.4)	20 (35.7)	2 (16.7)	
III	8 (42.1)	25 (44.6)	8 (66.7)	
IV	0 (0.0)	3 (5.4)	0 (0.0)	

Abbreviations: HCC, hepatocellular carcinoma; N, number.

a ANOVA test.

b Chi-square test.

In summary, our study revealed for the first time that L-FABP potently induces up-regulation of VEGF-A and increases angiogenic potential and migration activity in HCC cells. Our results also suggest that the function of L-FABP in HCC could be influenced by mutations in its cholesterol interaction sites. Together with previous reports, our findings indicate that L-FABP is a potential therapeutic target in HCC therapy.

## MATERIALS AND METHODS

### Subjects and tissues

Tumor and normal adjacent tissue (NAT) from 90 HCC patients were purchased from US Biomax, Inc. (array number: HLiv-HCC180Sur-02); the patients included 12 females and 78 males, with an average age of 53.5 ± 10.0 years (Table 3).

**Table 3:** Clinical characteristics of the cases included in analyses of L-FABP protein expression evaluated by immunohistochemistry

Characteristics	NAT, *N* = 90	HCC without cirrhosis, *N* = 57	HCC with cirrhosis, *N* = 33	P value
Age (years), Mean ± SD	53.5 ± 10.0	54.2 ± 10.3	52.2 ± 9.6	0.650 ^a^
Sex, N (%)				
Female	8 (13.3)	6 (10.5)	6 (18.2)	0.589 ^b^
Male	78 (86.7)	51 (89.5)	27 (81.8)	

Abbreviations: HCC, hepatocellular carcinoma; N, number.

a ANOVA test.

b Chi-square test.

c. One age miss data.

### Antibodies and chemical inhibitors

Antibodies specific to L-FABP, VEGF-A, flotillin-2, lamin A/C, α-tubulin, and β-actin were purchased from Santa Cruz Biotechnology (Texas, USA). Antibodies specific to VEGFR2, phospho-VEGFR2, proto-oncogene tyrosine-protein kinase Src (Src), phospho-Src, focal adhesion kinase 1 (FAK), phospho-FAK, phosphatidylinositol 3-kinase regulatory subunit (p85), RAC-alpha serine/threonine-protein kinase (Akt), phospho-Akt, serine/threonine-protein kinase mTOR (mTOR), phospho-mTOR, eukaryotic translation initiation factor 4E-binding protein 1 (4EBP1), phospho-4EBP1, and HIF-1α were obtained from Cell Signaling Technology. The chemical inhibitors Src inhibitor I and sorafenib were obtained from Calbiochem (Darmstadt, Germany) and Selleckchem (Boston, USA), respectively.

### Tissue microarray construction and immunohistochemistry

The tissue array sections were immunostained with specific antibodies against L-FABP (1:100) and VEGF-A (1:100). Pathologists at GenDiscovery Biotechnology, Inc. (Taipei City, Taiwan) interpreted the staining results, which were analyzed for the intensity and percentage of staining area by using Quick-score analysis, whereby scores (Q) were calculated as follows: Q = Percentage of positive cells (P) × Intensity (I); maximum Q = 300. The results were then graded according to the following criteria: 1: Q = 0–99, weak staining; 2: Q = 100–199, moderate staining; 3: Q = 200–299, strong staining; and 4: Q = 300, very strong staining.

### Cell culture

Huh7 and HepG2 cells were obtained from the Japanese Collection of Research Bioresources (National Institute of Health Sciences, Japan) and maintained in Dulbecco's modified Eagle's medium (DMEM) with 10% FBS. The immortalized cell line, which was derived from human primary hepatocytes, Hus-E/2 (Hus cells), was cultured in primary hepatocyte (PH) medium (DMEM containing 20 mM HEPES, 15 μg/ml L-proline, 0.25 μg/ml insulin, 50 nM dexamethasone, 44 mM sodium bicarbonate, 10 mM nicotinamide, 5 ng/ml EGF, and 0.1 mM ascorbic acid) with 10% FBS. All cell lines were incubated in a 5% CO2 atmosphere at 37°C.

### Creation and culture of L-FABP-overexpressed stable clones

To construct pcDNA3.1/L-FABP, a full-length L-FABP cDNA fragment (1–121 aa), which was cloned from the cDNA of Huh7 cells, was inserted into the pcDNA3.1 vector via the TOPO PCR cloning system (Life Technologies, NY, USA). The construct was validated using nucleotide sequencing. Hus cells were transfected with pcDNA3.1/L-FABP using Lipofectamine 2000 reagent (Invitrogen, NY, USA). After 2–4 weeks of culture in a medium containing 1 mg/ml G418 (Sigma-Aldrich, USA), stable clones were selected. Each clone was analyzed for L-FABP expression twice per month using western blots.

### Western blot analysis and immunoprecipitation

Proteins (50 μg) were resolved by 10% sodium dodecyl sulfate (SDS)-polyacrylamide gel electrophoresis and transferred to polyvinylidene fluoride membranes (Millipore, MA, USA). The membrane was first incubated with primary antibodies followed by horseradish peroxidase-conjugated secondary antibodies (Chemicon International). Signals were visualized using enhanced chemiluminescence detection reagent from Millipore, and the images were obtained using a Luminescence/Fluorescence Imaging System (LAS-4000; Fuji).

For immunoprecipitation, cell lysates containing 500 μg of protein were pre-cleared by protein A/G Sepharose beads (Millipore) and then incubated with anti-L-FABP or anti-VEGFR2 antibody overnight at 4°C. The immune complexes were washed three times in ice-cold PBS and subsequently captured by protein A/G Sepharose beads, and then the immunoprecipitated proteins were subjected to western blot analysis.

### Cell migration assay

To study 2D migration activity, cells were seeded on a 35-mm cell culture μ-Dish (ibidi, Planegg, Germany) at a density of 4×10^5^ cells per cm^2^ for a wound healing assay. Two days after seeding, the insert was removed with tweezers, yielding a standardized wound of 500 μm. The dish was washed with PBS and subsequently imaged for 0, 6, and 24 h.

To study 3D migration activity, cells were maintained in serum-free medium for 24 h and then seeded into Transwell Boyden chambers (Millipore). Subsequently, they were incubated in complete medium with 10% fetal bovine serum at 37°C for 16 h. The cells on the bottom side of the membrane were fixed with 1% formaldehyde/PBS for 15 min, stained with 0.1% crystal violet for 40 min, and then counted using an inverted contrast light microscope (Olympus CKX-41 with NIKON DSU-3 digital sight image system).

### Angiogenesis activity assay

#### Cell culture

Primary human umbilical vein endothelial cells (HUVECs) (Sciencell, CA, USA) were grown in M199 medium (containing 100 μg/ml Endothelial Cell Growth Supplement, 10 ng/ml heparin, and 5% fetal bovine serum) and cultured in a 5% CO2 atmosphere at 37°C.

#### In vitro tube formation assay

A 24-well plate was coated with 100 μl of Matrigel (1 mg/ml; BD Biosciences, CA, USA), which was allowed to solidify at 37°C for 1 h. HUVECs (1 × 10^4^ cells/well) were seeded on the Matrigel and incubated with conditioned medium collected from cultured cells (Huh7 cells: L-FABP-overexpressing or L-FABP-stable knockdown) for 8–12 h. The VEGF group was used to check the angiogenic activity of HUVEC cells. Photographs from random fields were acquired using a DP-50 microscope (Olympus, Tokyo, Japan), and each image was quantified according to the following formula [46]: Angiogenic score = [(No. of sprouting cells) × 1 + (No. of connected cells) × 2 + (No. of polygons) × 3)]/Total number of cells + [0, 1, or 2]. The definition of cell types and the parameters 0, 1, and 2 can be found in previous studies [9]. In brief, the presence of a complex mesh (luminal structures consisting of walls with a thickness of two to three cells) was given a score of 1. If this complex structure was present and the walls had a thickness of four or more cells thick, then a score of 2 was given. The absence of a complex mesh resulted in a score of 0 points.

#### In vivo Matrigel plug assay

Matrigel (phenol red-free; BD Biosciences) was mixed with L-FABP stable clones (2 × 10^6^ cells/Matrigel). The Matrigel plugs were subcutaneously injected into 10 4-week-old male NOD/SCID mice (one per mouse; see Animal Experiments below for details of mice) and recovered on Day 10 for analysis.

### Short interference RNA (siRNA) and short hairpin RNA (shRNA)

Modified oligonucleotides used as siRNA for L-FABP and control siRNA were obtained from Invitrogen. The shRNA clones were purchased from the National RNAi Core Facility Platform, Taiwan. For transfection, 1 × 10^5^ Hus/L-FABP or Huh7 cells were plated in a six-well plate for 24 h, and Lipofectamine 2000 was used to transfect siRNA or shRNA for knockdown of protein expression [47].

### Purification of L-FABP recombinant protein

L-FABP recombinant protein was purified using the Ni-NTA Purification system (Novex, USA) as previously described [48]. In particular, L-FABP-overexpressing Hus cells (transfected with the pcDNA3.1D/L-FABP plasmid) were lysed and sonicated in a native condition. After centrifugation, the supernatant was transferred to a fresh tube and allowed to slowly flow through the Ni-NTA resin by gravity. Subsequently, the resin was washed five times using Native Wash buffer (with 20 mM imidazole), eluted in 1 mL of Native Elution buffer (with 250 mM imidazole), and analyzed using SDS-PAGE (Supplementary Figure 4).

### Overlay assay (far-western blot)

The interaction between L-FABP and VEGFR2 (intracellular domain) was evaluated using the overlay assay as previous described [49]. To estimate the affinity of L-FABP and VEGFR2, an increased amount of VEGFR2 purified protein (#P3871, Abnova, USA) was loaded on SDS-PAGE and transferred to a PVDF membrane. After blocking, the membrane was washed and overlaid with V5-tagged L-FABP recombinant protein (1 μg/ml) prepared by the methods described above for 12 hours, followed by blotting with an anti-V5 antibody (Invitrogen) and HRP-conjugated secondary antibody. The signals were detected using enhanced chemiluminescence (ECL; Millipore) and recorded using a luminescence imaging system. Results were quantified by ImageJ software, and the affinity constant (Kd) of the interaction was determined by the non-linear regression fitting function of the GraphPad Prism 5 program (GraphPad Software).

### Lipid raft isolation

Raft microdomains were purified using a previously described method [50]. Briefly, 700 μl of 1% Triton X-100 lysis buffer was applied to pre-washed cells, and a Teflon-coated dounce homogenizer was used to disrupt the cell membranes (20–30 strokes). The lysate (4 mg) was incubated at 4°C for 30 min and mixed with an equivalent volume of 80% sucrose solution to yield a 40% sucrose gradient, and the mixture was transferred to a 12-ml polyallomer ultracentrifuge tube (suitable for an SW41 rotor) (Beckman Instruments). Subsequently, 6.5 and 3.5 ml of 30% and 5% sucrose cushion, respectively, was overlaid on the sample, and ultracentrifugation was applied at 187,813 *g* and 4°C for 20 h using an SW41 rotor. The floating opaque band corresponding to the detergent-resistant lipid rafts was collected and subjected to western blot analysis.

### Confocal microscopy analysis

L-FABP-stably expressing Hus cells were seeded onto a 22 × 22 mm cover slide, washed, fixed, and then permeabilized with 0.25% Triton X-100 for 10 min. For double-staining, the slides were first incubated with L-FABP and VEGFR2 primary antibodies overnight, then stained with Alexa488 (anti-mouse) and Alexa568 (anti-rabbit) (20 mU/mL) for 1 h in darkness, and finally counter-stained for nuclei with DAPI (10 ng/mL) for 10 min. The images were captured and analyzed using a Leica TCS SP5 Spectral Confocal Microscope (Leica Microsystems).

### Small GTPase binding assay

Cells (1 × 10^7^) were seeded and collected in 0.4 ml of ice-cold lysis buffer (50 mM Tris-HCl, pH 7.5, 10 mM MgCl2, 500 mM NaCl, 1% Triton X-100, and protease inhibitor cocktail). After lysing for 20 min on ice, cell debris was removed by centrifugation at 300 *g* and 4°C for 10 min. Half of each lysate (i.e., 50 μg of protein) was mixed with 15 μl of GST-PBD or GST-RBD beads, as recommended by a previous study [51], and incubated for 1 h at 4°C with rotation. In preparation for western blot analysis, samples were centrifuged (3,000 *g* for 1 min at 4°C), washed twice in ice-cold wash buffer (25 mM Tris-HCl, pH 7.5, 30 mM MgCl2, and 40 mM NaCl), and finally resuspended in 30 μl of SDS sample buffer and heated at 100°C for 5 min.

### Construction of human VEGF-A promoter

The VEGF-A promoter (full-length 1190 bp: from −1127 to +73) was synthesized by ShineGene Molecular Biotech Inc. (Shanghai, China) and constructed into the puc57 vector. Cutting of the full-length promoter with SacI and HindIII restriction enzymes allowed it to be cloned into the pGL4.22 luciferase reporter vector. The generated 5′ serial deletion constructs of the VEGF-A promoter, representing deletion of the HIF-1α binding site (the major transcription factor for regulation of VEGF-A expression), were named as follows: D1: bp −901 to +73; D2: bp −782 to +73; D3: bp −199 to +73. The primers used in the aforementioned cloning are listed in Supplementary Table 1. Nucleotide sequencing was used to validate all constructs.

### Luciferase reporter assay

L-FABP-overexpressing Hus cells were transfected with the constructed pGL4.22/VEGF-A promoter plasmids and the pGL4-Renilla luciferase control reporter plasmid as an internal control. After transfection with Lipofectamine 2000 and 24 h of incubation, the cells were lysed and luciferase activity was determined using the Dual-Luciferase Reporter Assay System (Promega) according to the manufacturer's protocol, in addition to a SpectraMax L luminometer (Molecular Devices, CA, USA).

### Chromatin immunoprecipitation assay

The chromatin immunoprecipitation assay was performed as follows. The cell lysate of Hus/L-FABP or control cells was sonicated, and then the chromatin was immunoprecipitated with HIF-1α antibody or rabbit immunoglobulin G antibody (Santa Cruz Biotechnology) as a negative control. After precipitation, the bound DNA was dissolved with 40 μl of ddH2O and then amplified by PCR with primers amplifying the HIF-1α binding element (−1041 to −750, Supplementary Table 1). The final PCR products were analyzed using 1.8 % agarose gels and visualized by ethidium bromide staining.

### Animal experiments

All animal experiments were conducted according to regulations approved by the Institutional Animal Care and Use Committee of College of Medicine, National Taiwan University. Male NOD-SCID mice (4 weeks old) were obtained from LASCO Taiwan Co., Ltd. For xenograft experiments, and Hus/L-FABP or Hus/Vector cells (2 × 10^6^ cells for each) were suspended in 200 μl of OPTI-MEM (Invitrogen) and inoculated into the right hindlimb of each mouse. Tumor size was measured twice per week with calipers, and the tumor volume was estimated using the following formula: (width)^2^ × length/2, as described in previous studies [52]. After 8 weeks, the mice were sacrificed and the tumors were removed, measured, and processed for immunohistochemistry.

To conduct a metastasis assay, we used a lung metastasis model described in previous studies [53]. Specifically, Hus/L-FABP or Hus/Vector cells (4 × 10^6^ cells each) were suspended in 100 μl of OPTI-MEM and introduced intravenously into the tail vein of male NOD/SCID mice. The mice were sacrificed after 10 weeks. Metastatic colonies in the lungs of mice were counted and photographed, and the removed lungs were fixed and embedded in paraffin for immunohistochemical analysis.

### Cloning of L-FABP mutants

The amino acid substitution of wild-type L-FABP protein was conducted as follows. L-FABP point-mutation clones, including Phe3 to Trp (F3W), Lys31 to Glu (K31E), and Thr94 to Ala (T94A), were generated using the QuickChange Site-Directed Mutagenesis Kit (Stratagene). The primers for PCR reactions and subsequent treatments with DpnI to eliminate the template DNA are listed in Supplementary Table 1. All constructs were validated using nucleotide sequencing.

### Statistical analysis

Relationships between protein expression and categorical variables were analyzed using chi-squared tests. For multivariate analysis, independent prognostic factors were determined using Cox's proportional hazard model. Survival curves were calculated using the Kaplan-Meier method and compared using log-rank tests. *In vitro* and *in vivo* experiments were analyzed in GraphPad Prism 5 (GraphPad Software), with the data presented as the mean ± standard error of the mean (SEM). Statistical significance was defined as *p* < 0.05.

## SUPPLEMENTARY FIGURES AND TABLES


