# Supplementary material for: Liver fatty acid-binding protein (L-FABP) promotes cellular angiogenesis and migration in hepatocellular carcinoma
Source: Oncotarget. 2016 Feb 22;7(14):18229–46. doi: 10.18632/oncotarget.7571 (PMC4951284; doi:10.18632/oncotarget.7571)
Supplement: Supplementary file 1 [file oncotarget-07-18229-s001.pdf]

SUPPLEMENTARY FIGURES AND TABLE

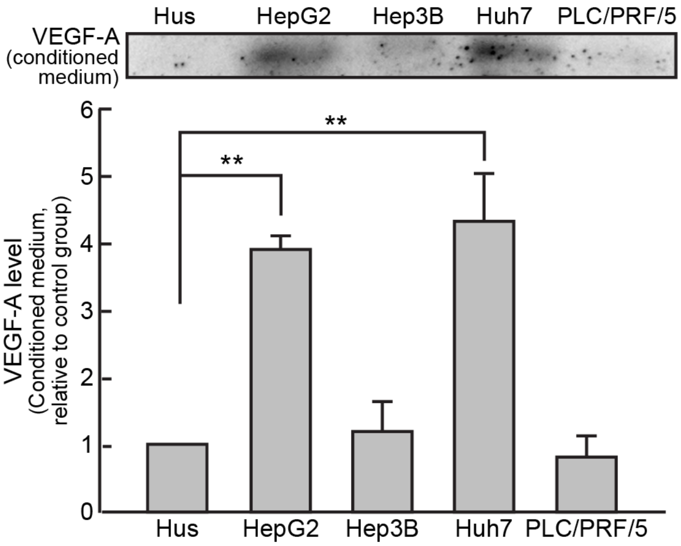

Supplementary Figure S1: Secreted VEGF-A was abundant in the culture medium of HepG2 and Huh7 cells. Culture medium of Hus, HepG2, Hep3B, Huh7, and PLC/PRF/5 cells was collected from a 10-cm dish (2×10^6 per dish for each cell line), and subjected to western blot analysis. **$p < 0.01$ versus Hus cells.

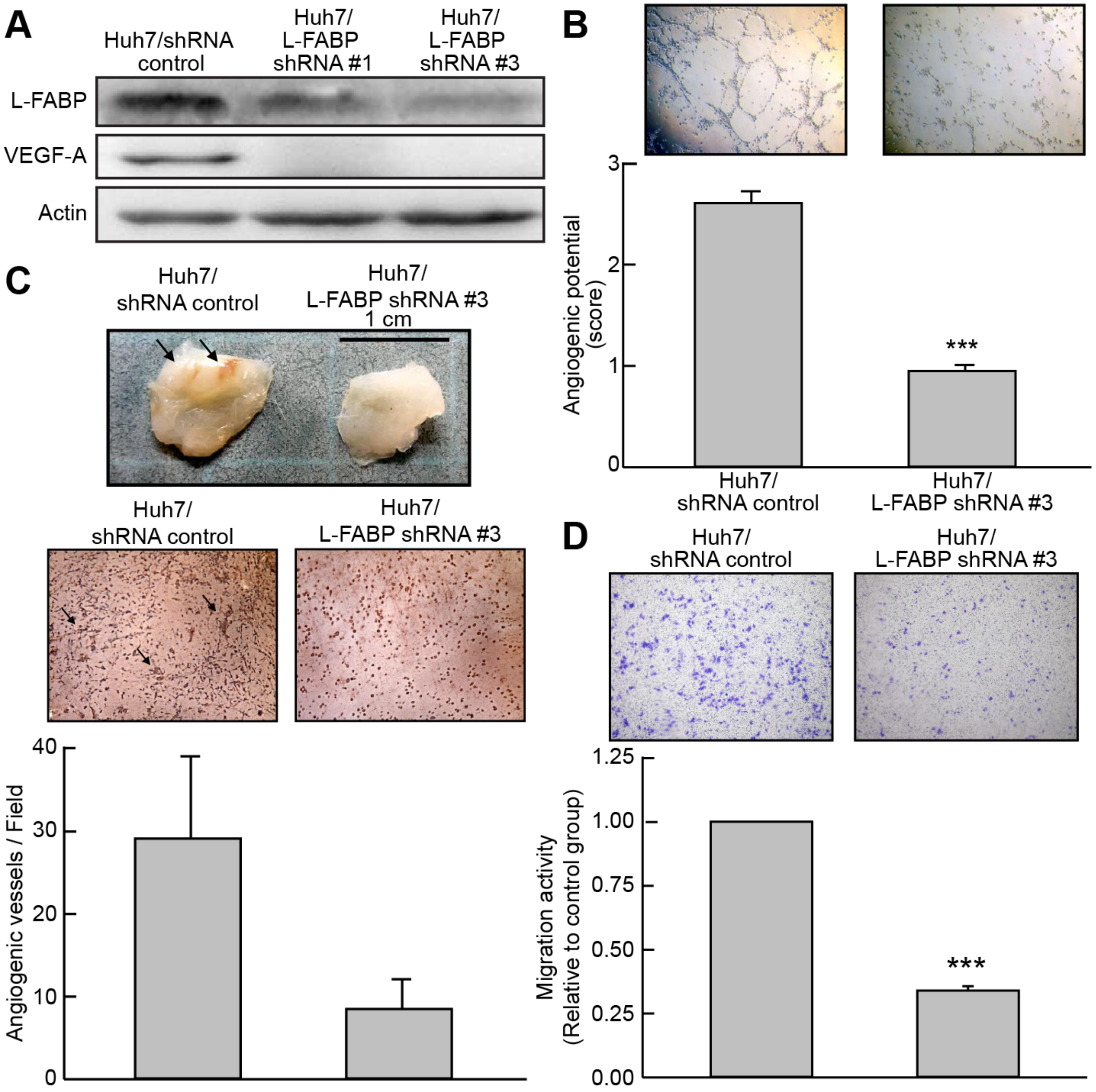

Supplementary Figure S2: Knockdown of L-FABP in Huh7 cells reduced VEGF-A expression, angiogenic activity, and migration. A. Western blot analysis of L-FABP and VEGF-A in Huh7 cells transfected with an shRNA control or L-FABP-targeting shRNA plasmids. Stable clones were screened and selected using puromycin. **B.** *In vitro* angiogenic activity (score: see *In vitro tube formation assay* in Methods for details) of L-FABP-stable knockdown Huh7 cells. Angiogenic vascular tubes were imaged at 8 h. ***p < 0.001 versus control. **C.** *In vivo* angiogenic activity in mice injected with Huh7/L-FABP shRNA or Huh7/shRNA control cells was assessed using a Matrigel plug assay. Left: Recovered Matrigel plugs. Arrows indicate the infiltration of blood vessels. Right: Immunohistochemical staining of CD31 (angiogenesis marker) in Matrigel plugs (n = 3, p = 0.067). **D.** 3D migration activity of Huh7/L-FABP shRNA stable clones seeded onto Boyden chambers and allowed to migrate for 16 h. ***p < 0.001 versus control.

KDR : 795~830 (aa)

```
                                            1                                                50
KDR kinase domian                     (1)   ILRTVKRANGGELKTGYLSIVMDPDELPLDEHCERLPYDASKWEFPRDRL
CD36 TSP binding domains              (1)   ------TYRVRFLAKENVTQDAEDNTVSFLQPNG----------------
DSLR domains                          (1)   -------GNCTHPEDMKLCTVIMKSNGSFPVNRRRV--------------
integrin alpha 1 cytoplasmic domains  (1)   -------WKIGFFKRPLKKKMEK---------------------------
integrin alpha 2 cytoplasmic domains  (1)   -------WKLGFFKRKYEKMTKNPDEIDETTELSS---------------
Consensus                             (1)          WKIGF KR  L  V     I
```

Supplementary Figure S3: Sequence alignment of FABP-interacting domains. Consensus sequence WKIGFXKRLXXVXXXI is likely to interact with L-FABP.

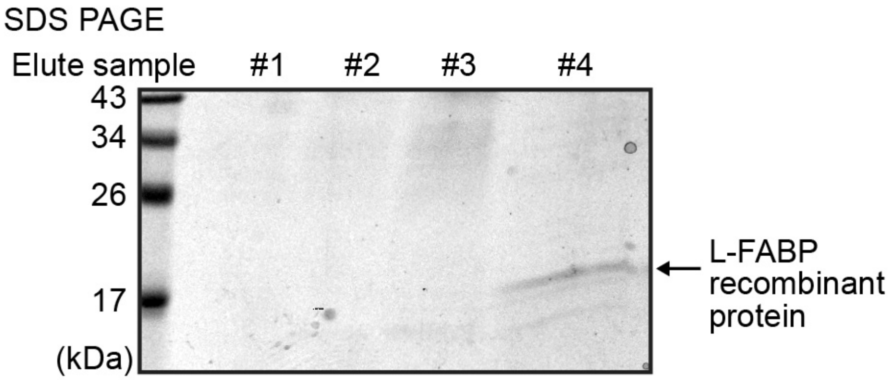

Supplementary Figure S4: Purification of L-FABP recombinant protein. L-FABP/V5-tagged recombinant protein was eluted by the fourth elution (with 250 mM imidazole) and subjected to SDS PAGE analysis. The size of the recombinant protein was nearly 20 kDa.

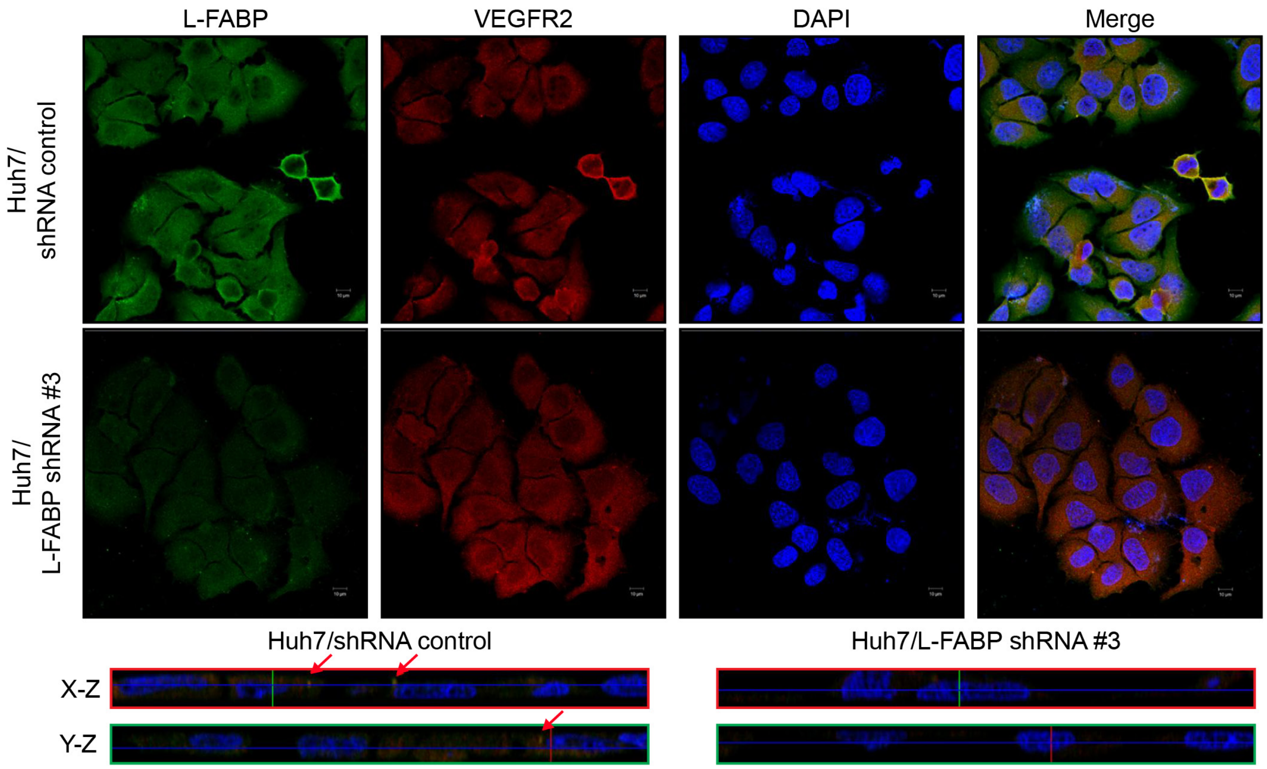

Supplementary Figure S5: Reduced co-localization of L-FABP and VEGFR2 on the membrane in Huh7 L-FABP-stable knockdown cells. A. Three-color confocal images of Huh7 L-FABP-stable knockdown cells fixed and stained with antibodies against VEGFR2 and L-FABP (magnification: 63×). Signals: green, L-FABP-Alexa 488; red, VEGFR2-Alexa 568; and blue, DAPI. The X-Z and Y-Z optical sections are shown for Huh7/shRNA control and Huh7/L-FABP shRNA cells in the lower photos. Arrows indicate co-localization of VEGFR2 and L-FABP on the apical membrane of cells.

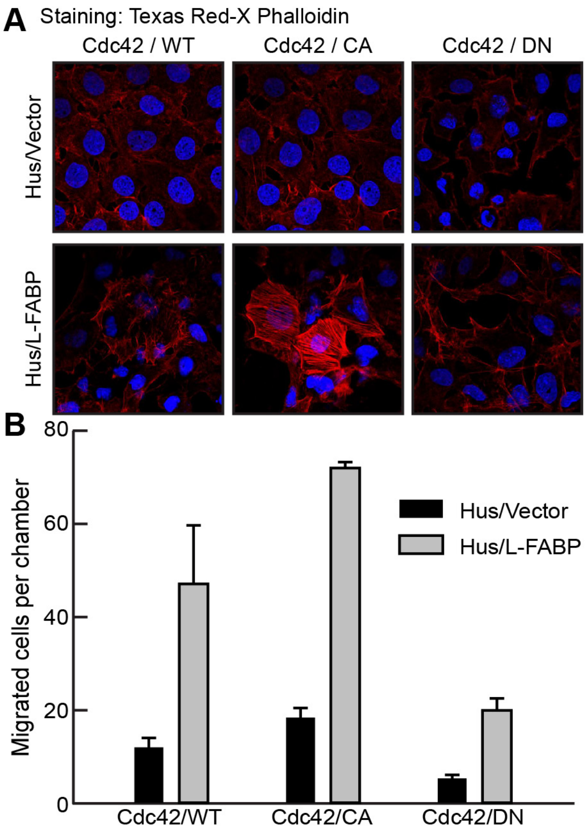

Supplementary Figure S6: L-FABP promoted actin rearrangement and migration activity of Hus cells via cdc42. Plasmids expressing different variants of cdc42, including wild-type (pRK-myc-Cdc42-wt, #12972), dominant negative (pRK-myc-Cdc42-T17N, #12973), and constitutively active (pRK-myc-Cdc42-Q61L, #12974) cdc42, were obtained from Addgene (Addgene, USA). Hus/L-FABP and control cells were transfected with these plasmids for 48 h, and immunofluorescence and transwell assays were performed. **A.** Cells were stained with Texas-Red-X phallodin to study the status of actin rearrangement. **B.** The migration activity of Hus/L-FABP cells was abolished by disruption of cdc42 activity.

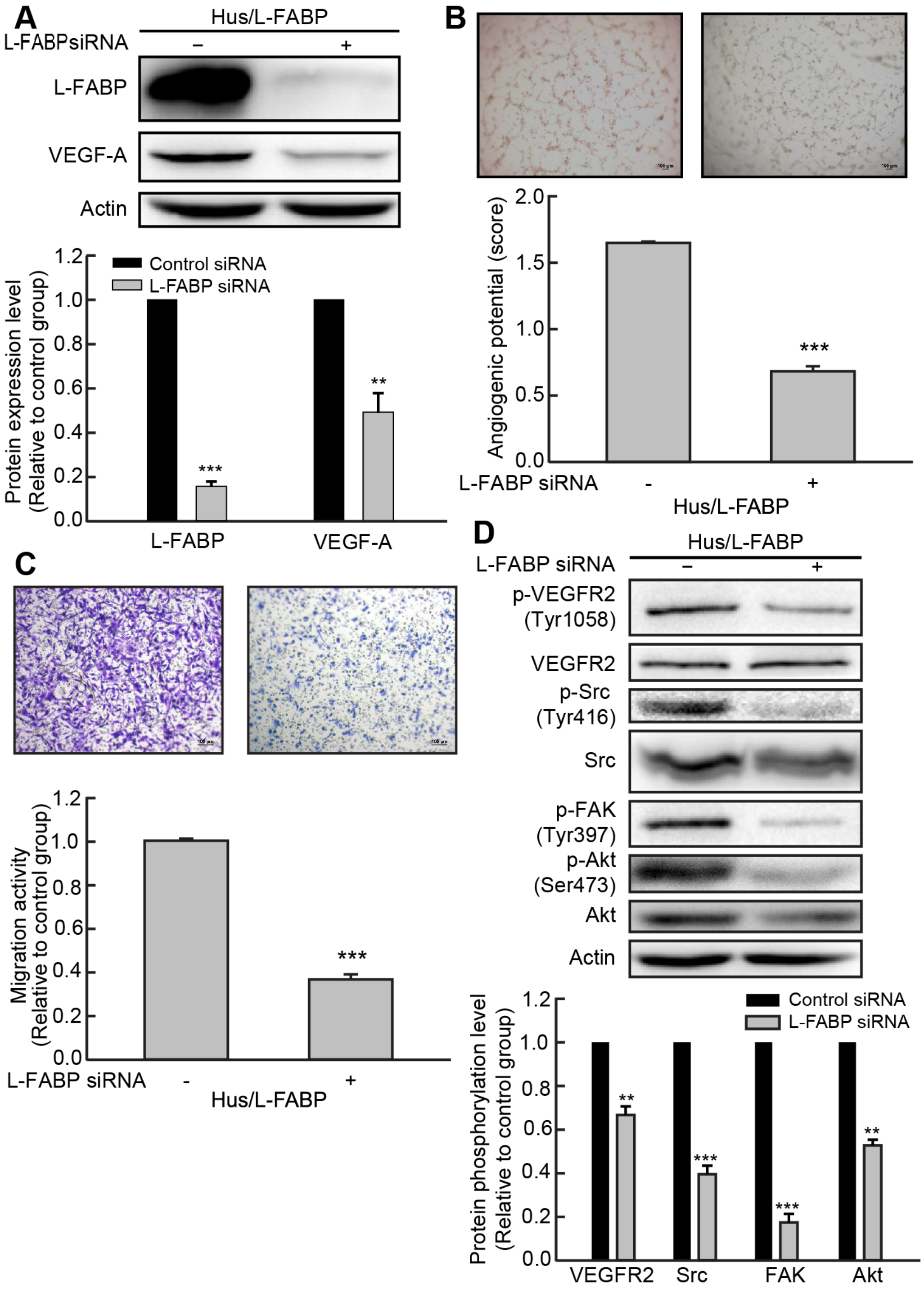

Supplementary Figure S7: Knockdown of L-FABP in Hus/L-FABP cells reversely reduced VEGF-A expression and migration activity. A. Western blot analysis of L-FABP and VEGF-A expression in Hus/L-FABP cells transfected with control siRNA or L-FABP-targeting siRNA for 24 h. $^{**}p < 0.01$, $^{***}p < 0.001$ versus control siRNA groups. **B.** *In vitro* angiogenic activity (score: see *In vitro tube formation assay* in Methods for details) in L-FABP siRNA-treated cells. Angiogenic vascular tubes wee imaged at 12 h. $^{***}p < 0.001$ versus control siRNA groups. **C.** To determine 3D migration activity, L-FABP siRNA-treated cells were seeded onto Boyden chambers and allowed to migrate for 16 h. $^{***}p < 0.001$ versus control siRNA groups. **D.** Western blot analysis showing phosphorylation of VEGFR2 (Tyr1058), Src (Tyr416), FAK (Tyr397), and Akt (Ser473) in Hus/L-FABP and Hus/Vector (vector-only control) cells. $^{**}p < 0.01$, $^{***}p < 0.001$ versus control siRNA groups.

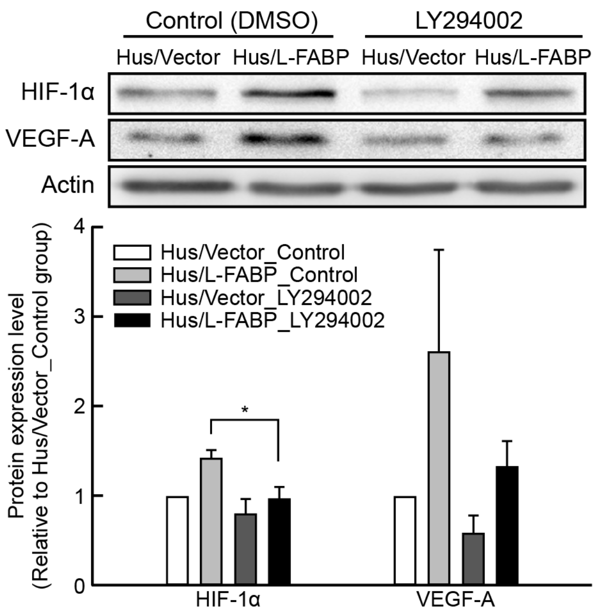

Supplementary Figure S8: Expression of HIF-1α and VEGF-A was regulated by the PI3K/Akt pathway. Hus/L-FABP and control cells were treated with LY294002 for 12 h and the lysates were subjected to western blot analysis. *$p < 0.05$ versus Hus/L-FABP control group.

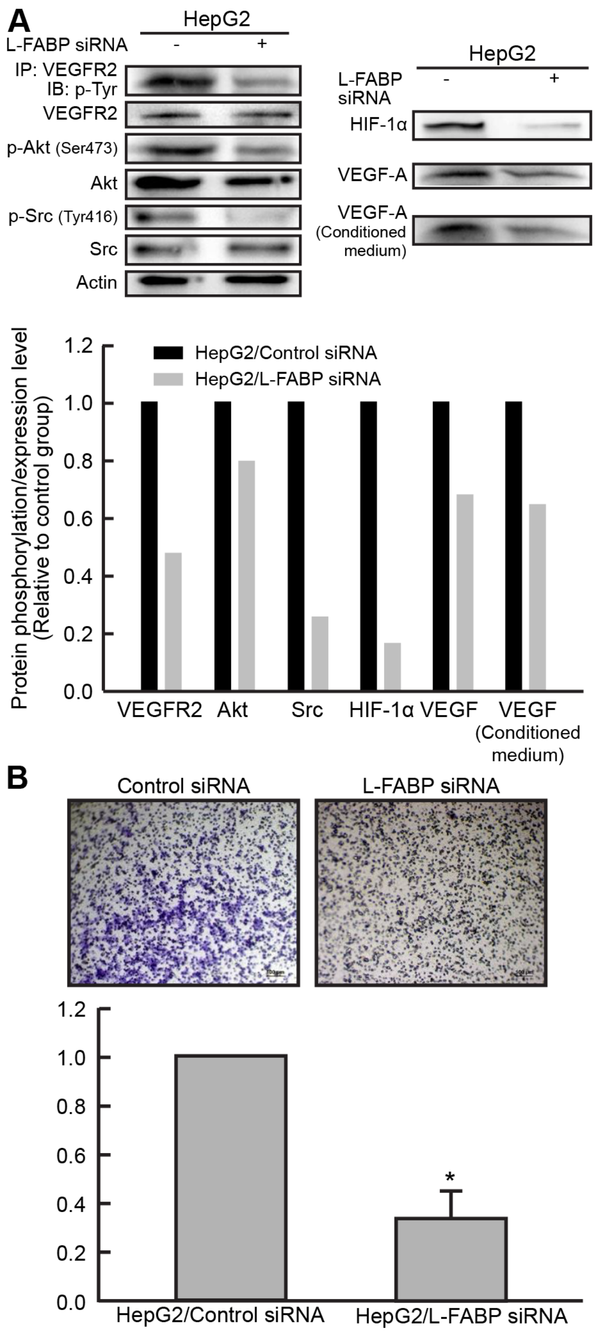

Supplementary Figure S9: Knockdown of L-FABP in HepG2 cells reduced VEGF-A expression and migration activity. **A.** Western blot analysis showed that the phosphorylation of VEGFR2 (Tyr1058), Akt (Ser473), and Src (Tyr416) and the expression of HIF-1α and VEGF-A were decreased in HepG2 cells transfected with L-FABP siRNA. **B.** To determine 3D migration activity, L-FABP siRNA-treated HepG2 cells were seeded onto Boyden chambers and allowed to migrate for 24 h. $*p < 0.05$ versus control siRNA groups.

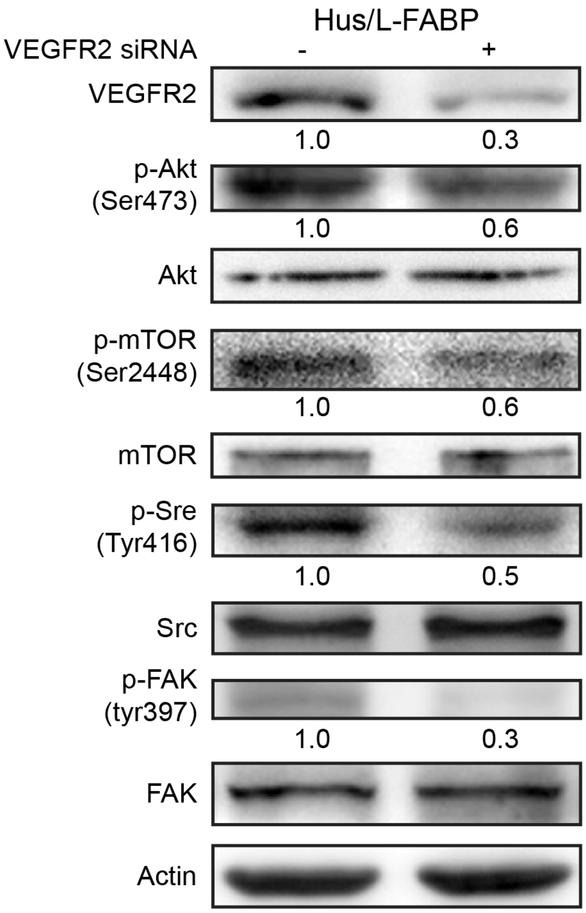

Supplementary Figure S10: Knockdown of VEGFR2 in Hus/L-FABP cells decreased the activation of downstream signaling molecules. Western blot analysis of the phosphorylation of signaling molecules including Akt, mTOR, Src, and FAK in Hus/L-FABP cells transfected with control siRNA or VEGFR2-targeting siRNA for 24 h.

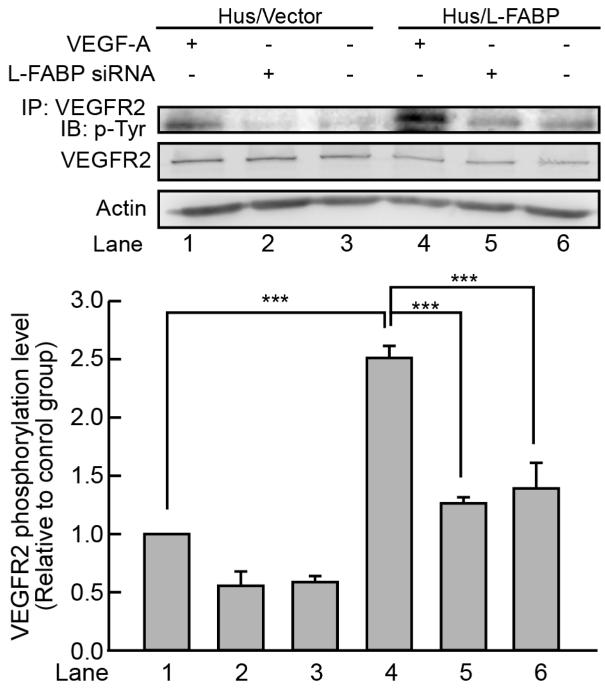

Supplementary Figure S11: L-FABP was required for promotion of the activation of VEGFR2. Hus/L-FABP and control cells were transfected with L-FABP siRNA or control siRNA for 24 h. After recovery, cells were then treated with VEGF-A (20 ng/ml, obtained from Sigma-Aldrich, USA) for 15 minutes, and immunoprecipitation and western blot analyses were performed (n=3, ***p < 0.001).

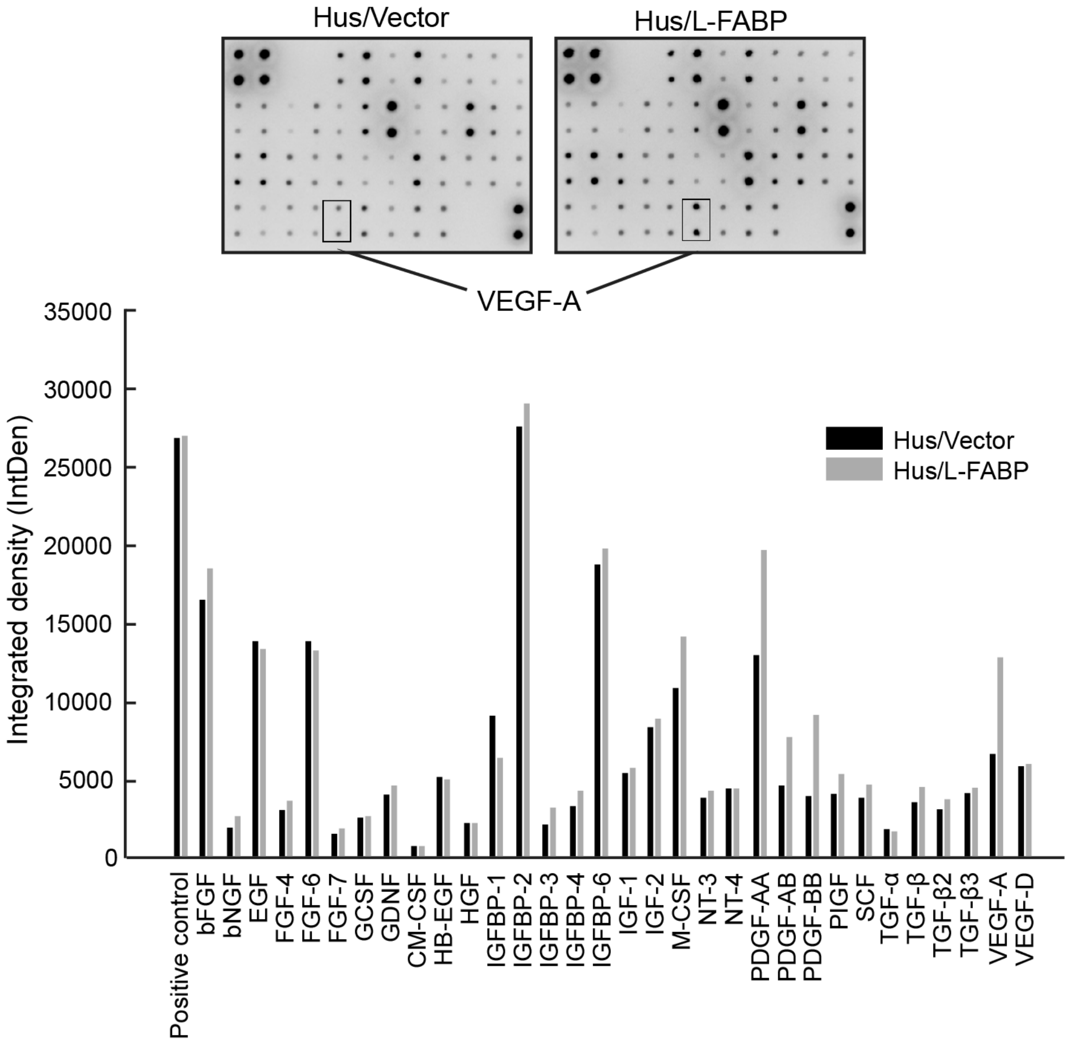

Supplementary Figure S12: Secreted VEGF-A levels in L-FABP-overexpressing cells were increased in human growth factor antibody array analysis. A human growth factor antibody array (#ab134002, Abcam, USA) was used to determine the amount of various growth factors secreted by L-FABP-overexpressing cells, and the results suggested that secreted levels of VEGF-A were elevated in Hus/L-FABP cells.

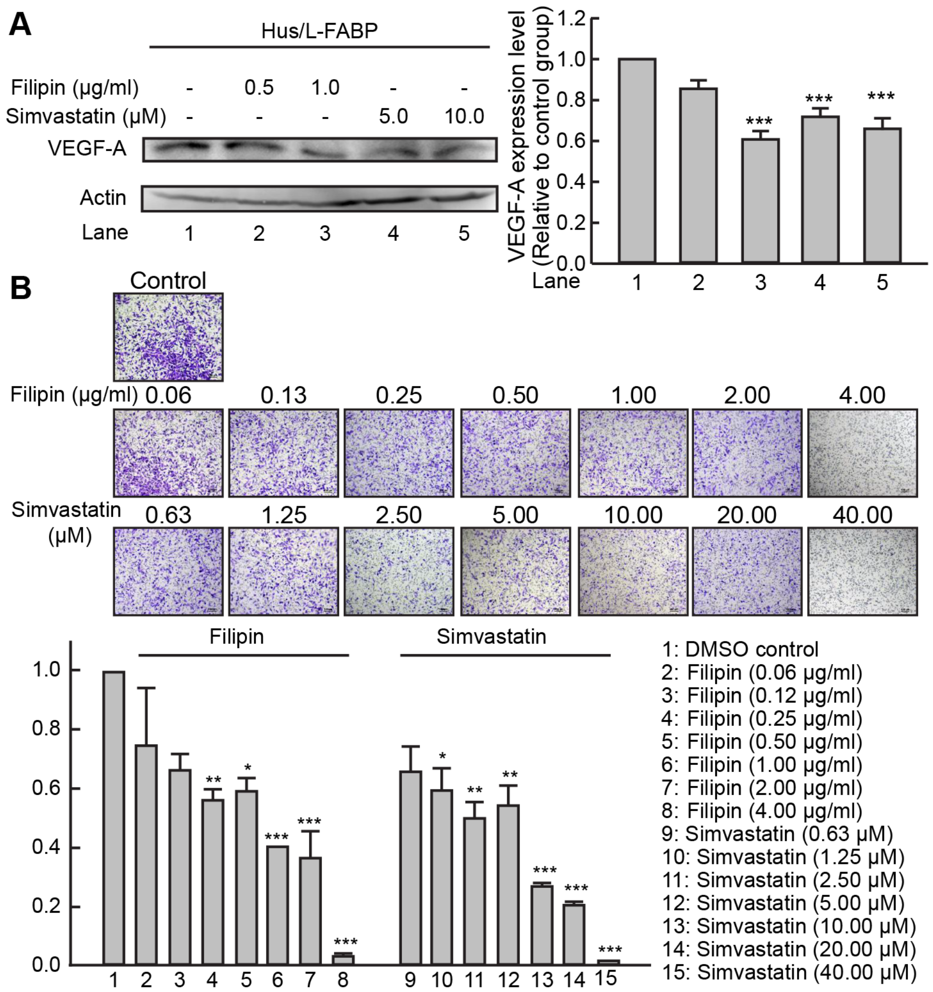

Supplementary Figure S13: Filipin and simvastatin significantly inhibited VEGF-A expression and migration activity of L-FABP-overexpressing cells. A. Western blot analysis of Hus/L-FABP cells treated with filipin (0.5 or 1.0 µg/ml) or simvastatin (5 or 10 µM) for 12 h. **p < 0.01, ***p < 0.001 versus DMSO-treated control group. **B.** Migration activity of Hus/L-FABP cells treated with various concentrations of fiipin and simvastatin for 12 h. *p < 0.05, **p < 0.01, ***p < 0.001 versus DMSO-treated control group.

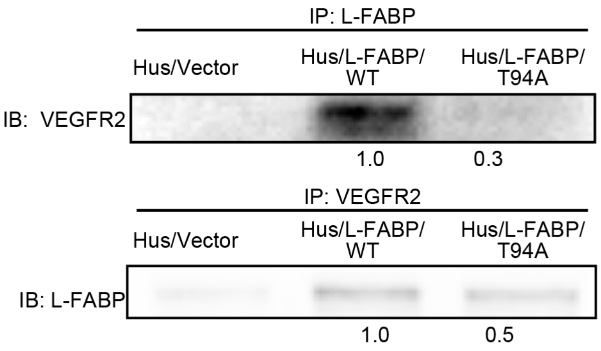

Supplementary Figure S14: T94A mutation of L-FABP disrupted the association with VEGFR2. Hus/L-FABP (wild-type), Hus/L-FABP (mutant, T94A), and Hus/Vector (vector-only control) cells were subjected to either immunoprecipitation (IP) with a VEGFR2 antibody followed by blotting with L-FABP or IP with an L-FABP antibody followed by blotting for VEGFR2.

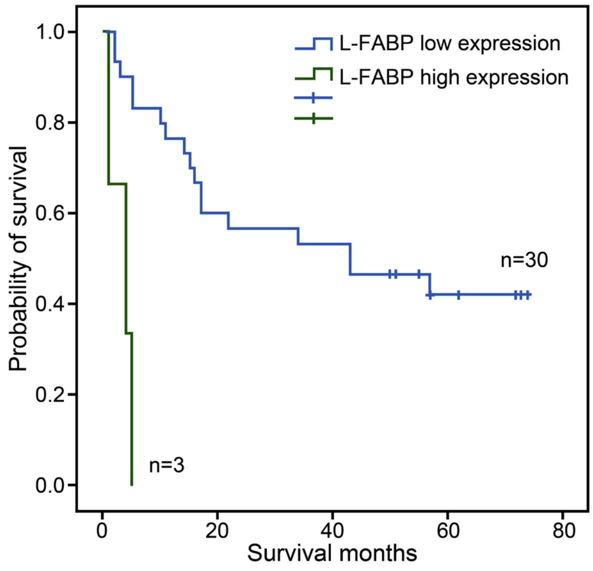

Supplementary Figure S15: Aberrant overexpression of L-FABP in HCC tissues (with cirrhosis) was associated with worse survival outcome. Kaplan-Meier survival curves demonstrate that the L-FABP high-expression group (n = 3) survived for fewer months than the L-FABP low-expression group (n = 30). ***p < 0.001.

Supplementary Table S1: List of primers, siRNA, and shRNA used in this study

Primers (5′ to 3′):

pcDNA 3.1/L-FABP cloning:
1. L-FABP TOPO PCR primer (Forward): CAC CAT GAG TTT CTC CGG CAA G
2. L-FABP TOPO PCR primer (Reverse): AAT TCT CTT GCT GAT TCT C

qRT-PCR:
1. L-FABP primer (Forward): ATG AGT TTC TCC GGC AAG TAC
2. L-FABP primer (Reverse): TCC TTC CCC TTC TGG ATG AGC
3. VEGF-A primer (Forward): CAT GAA CTT TCT GCT GTC TTG G
4. VEGF-A primer (Reverse): CCT GGT GAG AGA TCT GGT TCC
5. 18S rRNA primer (Forward): GCT TAA TTT GAC TCA ACA CGG GA
6. 18S rRNA primer (Reverse): AGC TAT CAA TCT GTC AAT CCT GTC

VEGF-A promoter cloning:
1. D1 primer (Forward): GGG GTA CCC CGC TCC ACA AAC TTG GTG CC
2. D2 primer (Forward): GGG GTA CCC CGA GGG CTC CAG ATG GCA
3. D3 primer (Forward): GGG GTA CCC CGT CGA GCT TCC CCT TCA TTG
4. Reverse primer(~+73): CCC TCG AGG GCG CCT CCC GAC AGA GCG CT

ChIP analysis (-1041 to -750):
Primer containing HIF-1α element (Forward):
CAG GAA CAA GGG CCT CTG TCT
Primer containing HIF-1α element (Reverse):
TGT CCC TCT GAC AAT GTG CCA TC

Site-directed mutagenesis cloning:
1. L-FABP F3W primer (Forward):
   ATG AGT TGG TCC GGC AAG TGG CAA CTG CAG
2. L-FABP F3W primer (Reverse):
   CTG CAG TTG CCA CTT GCC GGA CCA ACT CAT
3. L-FABP K31E primer (Forward):
   GAG CTC ATC CAG GAG GGG GAG GAT ATC AAG
4. L-FABP K31E primer (Reverse):
   CTT GAT ATC CTC CCC CTC CTG GAT GAG CTC
5. L-FABP T94A primer (Forward):
   CTG GTG ACA GCT TTC AAA AAC ATC
6. L-FABP T94A primer (Reverse):
   GAT GTT TTT GAA AGC TGT CAC CAG

siRNA & shRNA (5′ to 3′)
L-FABP siRNA (Invitrogen, FABP1HSS141976)
Primer number: 228624A01
(RNA)-GGU UCA GUU GGA AGG UGA CAA UAA A
Primer number: 228624A02
(RNA)-UUU AUU GUC ACC UUC CAA CUG AAC C

L-FABP shRNA (Acdemia Sinica, RNAi Core Lab)
Clone ID: TRCN0000059643
NM ID: NM_001443
Vector: pLKO.1
Target sequence: GTG ACA ATA AAC TGG TGA CAA
Hairpin sequence: CCGG-GTGACAATAAACTGGTGACAA-CTCGAG-TTGTCACCAGTTTATTGTCAC-TTTTTG
